# Anisocoria and an Array of Neurologic Symptoms in an Adult With Ewing Sarcoma

**DOI:** 10.6004/jadpro.2017.8.1.2

**Published:** 2017-01-01

**Authors:** Mary Jane LaRoche

**Affiliations:** University of Colorado Denver School of Medicine, Aurora, Colorado

## ABSTRACT

**CASE STUDY**

Ms. M, a 50-year-old female, reported a progressive 2-year history of sacral pain that became severe in the 2 to 3 months prior to her diagnosis of Ewing sarcoma (ES) and was initially evaluated by her gynecologist. Her presenting symptoms included urinary retention and impaired bowel function.

Evaluation via magnetic resonance imaging (MRI) revealed a 6-cm sacral mass consistent with a chordoma. Biopsy of the lesion led to the diagnosis of a high-grade small round blue cell tumor. High Ki67 index and CD-99 positivity supported a diagnosis of ES (Machado et al., 2009; Rocchi et al., 2010). The patient required urgent hospitalization for pain control, and repeat imaging demonstrated a 4-cm increase in the size of the sacral mass in the 1-month scan interval. Repeat biopsy was performed quickly, and chemotherapy was begun urgently when this biopsy revealed an EWSR1 translocation, confirming the diagnosis of ES (Machado et al., 2009).

No disease was found outside of the pelvis on imaging, and her ES was staged as grade 3, cT2b,Nx,Mx. For Ms. M, medical oncology recommended curative-intent chemotherapy with dose-dense CAV-IE (cyclophosphamide, doxorubicin [Adriamycin], vincristine, ifosfamide, and etoposide). The CAV-IE regimen alternates between CAV and IE every 14 days for 14 planned cycles, requiring growth factor support (Bacci et al., 2007; Jain & Kapoor, 2010; Womer et al., 2012). On day 4 of her first cycle of CAV chemotherapy treatment, Ms. M was noted to have some fine-motor tremors and dysmetria (inability to properly direct or limit motions), which were attributed to rapid upward titration of gabapentin for pain.

Ms. M also had worsening of her initial symptoms of sacral nerve compression during the initial hospital admission, requiring self-catheterization to urinate and digital stimulation to enable bowel movements. Radiation oncology was consulted. The goal of radiation therapy was definitive local control, with the hope of preserving and possibly improving bowel and bladder function.

After cycle 2 of IE, while receiving supportive hydration and antiemetics in the outpatient setting, Ms. M was found to have anisocoria (unequal pupil size; Figure 1), with a fully dilated, unreactive right pupil without ptosis (drooping of the upper eyelid), diplopia, pain or impaired extraocular movement, or other neurologic changes. The patient had significant nausea with emesis after her first treatment cycles and was on an extensive antiemetic regimen, including aprepitant, dexamethasone, ondansetron, prochlorperazine, lorazepam, and a scopolamine patch.

**Figure 1:**
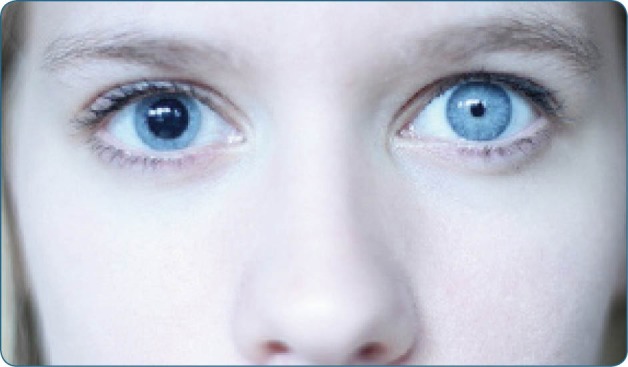
An example of anisocoria, showing the unequal pupil size.

Ms. M was sent to the emergency department for evaluation of the ansicoria. A brain computed tomography (CT) scan and neurologic exam were normal. Ms. M’s anisocoria was ultimately thought to be the result of an accidental topical exposure to the scopolamine during patch removal.

## ARTICLE

Ewing sarcoma (ES) is a rare mesenchymal malignancy that most commonly presents in adolescence as a primary bone cancer and less often as extraskeletal Ewing sarcoma (EES). Both ES and EES are part of a larger family of tumors that include small round blue cell tumors and Askin’s tumor of the chest wall. These malignancies share histologic, immunohistochemical characteristics, whereas ES is defined by the presence of a genomic translocation between EWSR1 and FLI1 (Harmon & Gebhardt, 2015).

The American Joint Committee on Cancer (AJCC) 7th Edition *Cancer Staging Manual* stages sarcoma based on tumor size, nodal involvement, and metastasis (TNM), along with a unique aspect of histologic grade (1–3). Histologic stage provides important information on sarcoma management (Massarweh, Dickson, & Anaya, 2015). Staging takes into account tumor size (< or > 5 cm) and depth (above superficial fascia without invasion of the fascia or deep, defined as exclusively beneath the superficial fascia, superficial to the fascia with invasion of or through the fascia; Edge et al., 2010). Nodal disease is staged similarly to other solid tumors. Metastatic disease can involve distant organs or arise within the same anatomic compartment (e.g., retroperitoneal sarcoma). Metastatic disease and histologic grade are important components of staging (Massarweh, Dickson, & Anaya, 2015).

Multidisciplinary team management is needed to deliver the multimodality care that is the cornerstone of treatment in both children and adults, including surgery, radiotherapy, and chemotherapy, which can benefit even those diagnosed with advanced disease (Siegel et al., 2015). Treatment decisions depend on sarcoma subtype, nonspecific histology, and staging (National Comprehensive Cancer Network [NCCN] Guidelines, 2016).

The oncology advanced practitioner is an important member of the sarcoma care team, from diagnosis to treatment and symptom management (Kurtin et al., 2015). Adult patients with the ES family of tumors are treated based on principles of treatment for pediatric populations, with more clinical trials in adult populations with this tumor type needed (Harmon & Gehhardt, 2015). In recent institutional studies, the 5-year overall survival rate for adults with this type of tumor was found to be between 46% and 60% (Ahmed, Robinson, Okuno, Rose, & Issa Laack, 2014; Bacci et al., 2007).

## POTENTIAL NEUROLOGIC SIDE EFFECTS OF EWING SARCOMA TREATMENT

What follows is a discussion of some of the potential neurologic side effects that may occur during cancer treatment and other unexpected neurologic symptoms experienced by a patient with ES.

Dysmetria (inability to properly direct or limit motions) and signs of cerebellar dysfunction have been most commonly attributed to the ifosfamide component of the CAV-IE chemotherapy regimen that Ms. M received. Ifosfamide is also associated with a 12% to 15% incidence of central nervous system (CNS) toxicity, although an incidence of 30% may be reported (Lexicomp Online, 2016a). Ms. M did not develop any CNS toxicity or encephalopathy related to the ifosfamide. Central nervous system toxicity can lead to discontinuation of ifosfamide and may require the use of methylene blue or other agents to reverse the encephalopathy (Pelgrims et al., 2000).

A variety of neurologic complications can be associated with components of CAV-IE, including headache, acute encephalopathy, vasculopathy and stroke, acute cerebellar syndrome, peripheral neuropathy and optic neuritis, as well as transient cortical blindness (Lee & Wen, 2016).

During her first cycle of the IE component of chemotherapy, Ms. M developed horizontal nystagmus without vision changes, initially attributed to prochlorperazine or opioids. Extrapyramidal side effects, including pseudoparkinsonism, have been described with prochlorperazine, with a report of oculogyric crisis (involuntary upward gaze; Lexicomp Online, 2016b). Nystagmus has been reported in < 1% of patients receiving morphine and also is noted with other opioids such as hydromorphone (Lexicomp Online, 2016c, 2016d). Opioids and prochlorperazine have anticholinergic effects, which can manifest as blurred vision (Lexicomp Online, 2016b).

To evaluate nystagmus, a brain MRI was considered but postponed due to the patient’s claustrophobia, with open MRI available only in the outpatient setting. Brain metastasis is found in less than 5% of bone and soft-tissue sarcomas (Shweikeh et al., 2014). Fortunately, the patient’s nystagmus resolved shortly after hospital discharge and did not recur. She has since tolerated prochlorperazine without any further side effects.

**Anisocoria**

Anisocoria can present more commonly in the oncology setting as a Pancoast tumor or superior sulcus tumor in apical lung cancers with Horner syndrome. Horner syndrome manifests with classic neurologic symptoms of miosis (constriction of the pupil), ptosis, and anhidrosis (absence of sweating) due to involvement of the sympathetic pathways that supply the head, eye, and neck, and it has a reported prevalence of 14% to 50% (Arcasoy, Jett, & Schild, 2013).

Anisocoria can be indicative of impaired dilation or constriction of one pupil and may represent a benign symptom or herald a neurologic emergency from trauma, stroke, thrombosis, or tumor. Approximately 20% of the population has anisocoria of 0.4 mm (Lam, Thompson, & Corbett, 1987). Looking at old photos may help to identify a previously unrecognized anisocoria. The late musician David Bowie had permanent anisocoria following an injury that paralyzed the muscle that constricted the pupil in his left eye (Basu, 2016).

The size of the pupil is controlled by the action of two opposing iris muscle groups: the dilator and sphincter pupillae. Constriction of the pupil is mediated by parasympathetic (cholinergic) nerves and dilation by the sympathetic pathway. If anisocoria is greater when examination takes place in a darkened setting, a small pupil is abnormal, possibly representing a sympathetic lesion. Alternatively, if the pupil is larger when there is more light, a larger pupil is abnormal, possibly representing a parasympathetic lesion.

*Possible Etiologies*: Pharmacologic mydriasis (dilation of the pupil) is known to occur with medications given for ocular conditions, including parasympathetic cycloplegic drugs such as atropine and sympathomimetic drugs including phenylephrine, clonidine, and apraclonidine. Unintentional anisocoria has been reported in the literature with aerosolized ipratropium due to poorly fitting masks (Bisquerra, Botz, & Nates, 2005; Iosson, 2006; Jannun & Mickel, 1986; Openshaw, 2006; Wehbe, Antoun, Moussa, & Nassif, 2008) and with plants such as jimsonweed and angel trumpet due to their anticholinergic properties (Firestone & Sloane, 2007; Macchiaiolo et al., 2010; Savitt, Roberts, & Siegel, 1986).

In addition, anisocoria has been reported in the emergency room with unintended eye contact after use of a hemorrhoidal cream (Polomský & Smereck, 2012). Anisocoria has also been reported with selective serotonin-reuptake inhibitors (Barrett, 1994; Falavigna, Kleber, Teles, & Molossi, 2010) and diphenhydramine (Delaney, 1980). Finally, anisocoria has been reported with scopolamine patches (Bienia, Smith, & Pelligrino, 1983; Lee et al., 2013; Lin, 2001; McCrary, & Webb, 1982; Price, 1985), which are utilized not only for motion sickness, but for refractory nausea and vomiting in the oncology setting.

**Figure 2:**
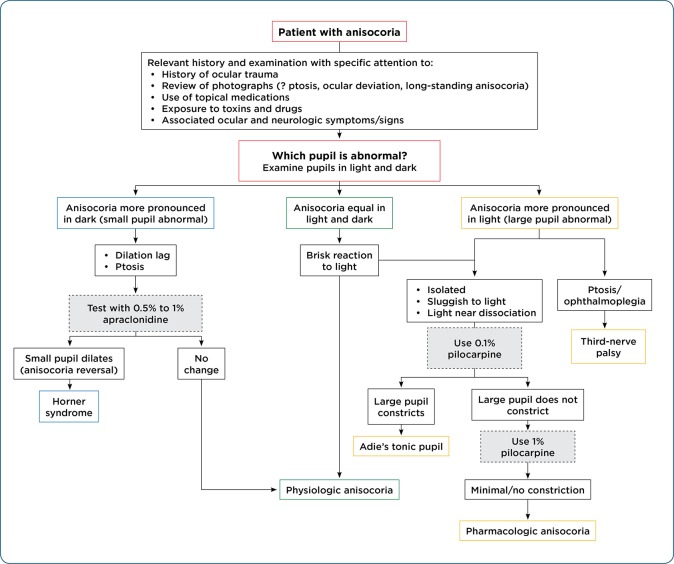
Flowchart illustrating the approach to a patient with anisocoria.

*Assessment Approach*: Evaluation of anisocoria included a brain computed tomography (CT) scan and neurologic exam, which were normal for Ms. M. Brain tumors are relatively uncommon in ES; however, thrombocytopenia can occur with CAV-IE. Therefore, intracranial hemorrhage is a consideration upon a finding of anisocoria. Ms. M’s anisocoria was ultimately thought to be a result of accidental topical exposure to the scopolamine during patch removal. Use of gloves by patients and staff prior to the application and removal of patches and other topical agents may help to avoid accidental exposure. Algorithms for the evaluation of anisocoria are available (see Figure 2; Kedar, Biouse, & Newman, 2015).

## SUMMARY

Ms. M presented with an aggressive but potentially curable ES with a variety of symptoms, which led to a delay in diagnosis and an emergency admission for worsening bowel and bladder dysfunction (Widhe & Widhe, 2000). Although she did not experience encephalopathy, a worrisome neurologic side effect associated with ifosfamide and the CAV-IE regimen, Ms. M manifested a variety of neurologic symptoms and side effects requiring meticulous care from her multidisciplinary team, most notably, her advanced practitioners. Sacral nerve compression due to the location of her ES caused urinary and bowel function impairment. Supportive medications also contributed to temporary neurologic symptoms.

In this case, the sudden onset of anisocoria was due to unintentional ocular exposure from a scopolamine patch. As of the writing of this article, Ms. M is nearing completion of the 14 planned cycles of CAV-IE and has no evidence of disease on recent imaging. Her sacral pain has resolved, and side effects from chemotherapy have been well managed in recent cycles. Ms. M has accepted that her bowel and bladder function will not recover. The care team remains vigilant in supporting Ms. M with her complex care needs while continuing to monitor her for treatment-related side effects and anticipate her future surveillance and survivorship needs.
